# Racial Discrimination in Healthcare and Public Settings as a Predictor of Suicidal Ideation Onset: Insights from a National Longitudinal Study

**DOI:** 10.1192/j.eurpsy.2026.10467

**Published:** 2026-07-31

**Authors:** S. Kim, K. P. Fung

**Affiliations:** 1 Psychiatry and Behavioural Neurosciences, McMaster University, Hamilton; 2 Psychiatry, University of Toronto, Toronto, Canada

## Abstract

**Introduction:**

Suicidal ideation constitutes a critical precursor to suicide attempts and deaths. While risk factors such as age, sex, and socioeconomic status have been studied extensively, the role of perceived racial discrimination (RD)—particularly within healthcare contexts—remains insufficiently explored.

**Objectives:**

The study aims to determine whether perceived RD in healthcare and public settings independently predicts the onset of suicidal ideation (SI) among U.S. adults with depressive symptoms, while accounting for established sociodemographic risk factors.

**Methods:**

This investigation utilizes data from Waves 1 and 2 of the National Epidemiologic Survey on Alcohol and Related Conditions (NESARC), with an analytic sample of 2,123 adults aged 18 years and older. Participants were selected based on a history of depressed mood or anhedonia and were free of SI at baseline. RD was assessed via self-report in healthcare and public settings and categorized dichotomously. Multivariate logistic regression models were used to examine associations between RD, sociodemographic characteristics, and new-onset SI at three-year follow-up.

**Results:**

Perceived RD in healthcare settings significantly increased the odds of SI onset after adjustment for confounders, while RD in public settings did not remain significant following multivariate adjustment. Additional independent risk factors for SI onset included male sex, younger age groups, lower educational attainment, low and middle household income, and Asian/Native Hawaiian/Other Pacific Islander ethnicity. Notably, even after accounting for RD and structural factors, Asian identity remained associated with substantially greater risk.

**Conclusions:**

Perceived discrimination within healthcare is a significant, independent predictor of suicidal ideation among adults with depressive symptoms. As disparities persisted particularly for Asian populations, these findings underscore the need for mental health system interventions that address provider bias and structural inequities, and suggest that culturally responsive, anti-racist measures in healthcare could play a pivotal role in suicide prevention for marginalized groups

**Disclosure of Interest:**

None Declared

